# Ethyl 3-(4-fluoro­phen­yl)-6-methyl-4-oxo-2-(1-cyclohexylamino)-3,4-dihydro­furo[2,3-*d*]pyrimidine-5-carboxyl­ate

**DOI:** 10.1107/S1600536807066780

**Published:** 2007-12-21

**Authors:** Yong Sun, Guo-Ping Zeng, Yang-Gen Hu

**Affiliations:** a Yunyang Teachers College, Danjiangkou 442700, People’s Republic of China; b Department of Chemistry and Life Science, Hubei University of Education, Wuhan 430205, People’s Republic of China; c Key Laboratory of Pesticides and Chemical Biology, Ministry of Education, Central China Normal University, Wuhan 430079, People’s Republic of China; d Department of Medicinal Chemistry, Yunyang Medical College, Shiyan 442000, People’s Republic of China

## Abstract

In the crystal structure of the title compound, C_22_H_24_FN_3_O_4_, the two fused rings of furo[2,3-*d*]pyrimidine form a dihedral angle of 0.88 (13)°. The attached benzene ring is twisted with respect to the heterocyclic pyrimidinone ring, making a dihedral angle of 75.07 (12)°. The cyclo­hexyl ring shows a distorted chair conformation. The mol­ecular structure is stabilized by intra­molecular C—H⋯O and C—H⋯N hydrogen-bonding inter­actions. The crystal packing is mainly stabilized by C—H⋯π hydrogen-bond inter­actions. Further stability is provided by C—F⋯π and C—O⋯π stacking inter­actions.

## Related literature

The preparation and biological activity are described by Miyazaki *et al.* (2007 ▶), Gangjee *et al.* (2006 ▶) and Lagu *et al.* (2000 ▶). For related literature, see: Ding *et al.* (2004 ▶). For the crystal structure of another fused pyrimidinone derivative, see: Hu *et al.* (2007 ▶).
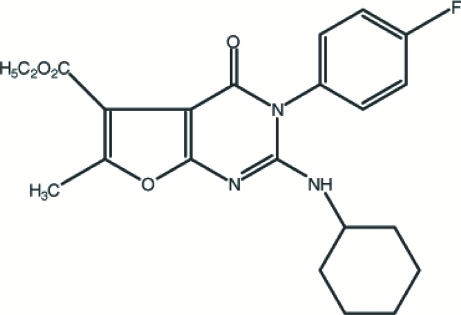


## Experimental

### 

#### Crystal data

C_22_H_24_FN_3_O_4_
*M* *_r_* = 413.44
Triclinic, 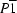

*a* = 9.2051 (8) Å
*b* = 10.7957 (9) Å
*c* = 11.6601 (10) Å
α = 106.681 (1)°
β = 100.417 (2)°
γ = 101.550 (2)°
*V* = 1051.85 (16) Å^3^
*Z* = 2
Mo *K*α radiation
μ = 0.10 mm^−1^
*T* = 291 (2) K
0.30 × 0.30 × 0.20 mm

#### Data collection

Bruker SMART 4K CCD area-detector diffractometer
Absorption correction: multi-scan (*SADABS*; Sheldrick, 2003 ▶) *T*_min_ = 0.972, *T*_max_ = 0.981
10785 measured reflections
4505 independent reflections
2941 reflections with *I* > 2σ(*I*)
*R*_int_ = 0.036

#### Refinement

*R*[*F* ^2^ > 2σ(*F* ^2^)] = 0.066
*wR*(*F* ^2^) = 0.182
*S* = 1.09
4505 reflections
276 parameters
H atoms treated by a mixture of independent and constrained refinement
Δρ_max_ = 0.27 e Å^−3^
Δρ_min_ = −0.21 e Å^−3^

### 

Data collection: *SMART* (Bruker, 2001 ▶); cell refinement: *SAINT-Plus* (Bruker, 2001 ▶); data reduction: *SAINT-Plus*; program(s) used to solve structure: *SHELXS97* (Sheldrick, 1997 ▶); program(s) used to refine structure: *SHELXL97* (Sheldrick, 1997 ▶); molecular graphics: *PLATON* (Spek, 2003 ▶); software used to prepare material for publication: *SHELXTL* (Sheldrick, 2001 ▶).

## Supplementary Material

Crystal structure: contains datablocks I, global. DOI: 10.1107/S1600536807066780/at2523sup1.cif

Structure factors: contains datablocks I. DOI: 10.1107/S1600536807066780/at2523Isup2.hkl

Additional supplementary materials:  crystallographic information; 3D view; checkCIF report

## Figures and Tables

**Table 1:** Hydrogen-bond and C—F⋯π and C—O⋯π interactions (Å, °) *Cg*2 and *Cg*3 are the centroids of the N1/C9/C7/C8/N2/C10 and C11–C16 rings, respectively.

*D*—H⋯*A*	*D*—H	H⋯*A*	*D*⋯*A*	*D*—H⋯*A*
C17—H17⋯N2	0.98	2.41	2.813 (3)	104
C6—H6*A*⋯O2	0.96	2.45	3.039 (3)	120
C20—H20*B*⋯*Cg*3^i^	0.97	2.97	3.820 (4)	147
C14—F1⋯*Cg*3^ii^	1.36 (1)	3.36 (1)	3.732 (3)	95
C3—O2⋯*Cg*2^iii^	1.21 (1)	3.31 (1)	3.409 (3)	84

**Fig. 1.**
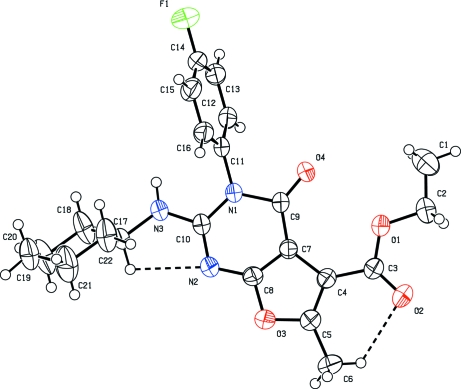
The molecular structure of the title compound (I), showing the atom-labeling scheme. Displacement ellipsoids are drawn at the 50% probability level. Only the intramolecular C—H···O and C—H···N hydrogen bonds is shown as dashed lines.

**Fig. 2.**
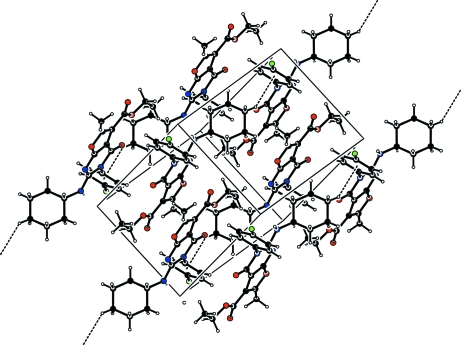
A view of the packing and hydrogen bonding interactions of (I).

## supplementary crystallographic information

### Comment

Fused pyrimidine compounds are valued not only for their rich and varied chemistry, but also for many important biological properties. Among them, the furopyrimidine ring system, because of a formal isoelectronic relationship with purine, is of special biological interest (Miyazaki *et al.*, 2007; Gangjee *et al.* 2006; Lagu *et al.*, 2000). Recently, we have focused on the synthesis of the heterocycle systems containing fused furopyrimidine *via* aza-Wittig reaction at room temperature (Hu,*et al.*, 2007). Herein, we present X-ray crystallographic analysis of the compound (I) in this paper,(Fig. 1), which may be used as a new precursor for obtaining bioactive molecules.

In the molecule (I), the bond lengths and angles are unexceptional. The two fused rings of furo[2,3-*d*]pyrimidine form a dihedral angle of 0.88 (13)°. The C11—C16 phenyl ring is twisted with respect to pyrimidinone ring, with a dihedral angle of 75.07 (1)°. The cyclohexyl ring in (I) shows a distored chair conformation [φ =30.0 (3)° and θ = 2.5 (3)°, puckering amplitude = 0.557 (3) Å]. The molecular structure is stabilized by intramolecular C—H···O and C—H···N hydrogen bonds interactions (Table 1). The crystal packing is mainly stabilized by C—H···π hydrogen bonding interactions. Further stability is provided by C—F···π and C—O···π stacking interactions.

### Experimental

To a solution of the diethyl 2-((4-fluorophenylimino)methyleneamino)-5-methylfuran- 3,4-dicarboxylate (3 mmol) in dichloromethane (5 ml) was added cyclohexanamine (3 mmol). After stirring the reaction mixture for 1 h, the solvent was removed and anhydrous ethanol (10 ml) with several drops of EtONa in EtOH was added. The mixture was stirred for 3 h at room temperature. The solution was concentrated under reduced pressure and the residue was recrystallized from ethanol to give the title compound in a yield of 84%. Crystals suitable for single-crystal X-ray diffraction were obtained by recrystallization from a mixed solvent of ethanol and dichloromethane (1:1 *v*/*v*) at room temperature.

### Refinement

All H atoms were located in difference maps and treated as riding atoms, with N—H = 0.86 Å and C—H = 0.93 - 0.98 Å, and *U*_iso_ = 1.2 or 1.5*U*_eq_ (C,*N*).

### Crystal data

**Table:**

C_22_H_24_FN_3_O_4_	*Z* = 2
*M**_r_* = 413.44	*F*_000_ = 436
Triclinic, *P*1	*D*_x_ = 1.305 Mg m^−^^3^
Hall symbol: -P 1	Mo *K*α radiation λ = 0.71073 Å
*a* = 9.2051 (8) Å	Cell parameters from 1930 reflections
*b* = 10.7957 (9) Å	θ = 2.3–22.7º
*c* = 11.6601 (10) Å	µ = 0.10 mm^−^^1^
α = 106.681 (1)º	*T* = 291 (2) K
β = 100.417 (2)º	Block, colourless
γ = 101.550 (2)º	0.30 × 0.30 × 0.20 mm
*V* = 1051.85 (16) Å^3^	

### Data collection

**Table:**

Bruker SMART 4K CCD area-detector diffractometer	4505 independent reflections
Radiation source: fine-focus sealed tube	2941 reflections with *I* > 2σ(*I*)
Monochromator: graphite	*R*_int_ = 0.036
*T* = 291(2) K	θ_max_ = 27.0º
φ and ω scans	θ_min_ = 1.9º
Absorption correction: multi-scan(SADABS; Sheldrick, 2003)	*h* = −11→11
*T*_min_ = 0.972, *T*_max_ = 0.981	*k* = −13→13
10785 measured reflections	*l* = −14→14

### Refinement

**Table:**

Refinement on *F*^2^	Secondary atom site location: difference Fourier map
Least-squares matrix: full	Hydrogen site location: inferred from neighbouring sites
*R*[*F*^2^ > 2σ(*F*^2^)] = 0.066	H atoms treated by a mixture of independent and constrained refinement
*wR*(*F*^2^) = 0.182	*w* = 1/[σ^2^(*F*_o_^2^) + (0.084*P*)^2^ + 0.0618*P*] where *P* = (*F*_o_^2^ + 2*F*_c_^2^)/3
*S* = 1.09	(Δ/σ)_max_ = 0.001
4505 reflections	Δρ_max_ = 0.27 e Å^−^^3^
276 parameters	Δρ_min_ = −0.20 e Å^−^^3^
Primary atom site location: structure-invariant direct methods	Extinction correction: none

### Special details

**Table:**

Geometry. All e.s.d.'s (except the e.s.d. in the dihedral angle between two l.s. planes) are estimated using the full covariance matrix. The cell e.s.d.'s are taken into account individually in the estimation of e.s.d.'s in distances, angles and torsion angles; correlations between e.s.d.'s in cell parameters are only used when they are defined by crystal symmetry. An approximate (isotropic) treatment of cell e.s.d.'s is used for estimating e.s.d.'s involving l.s. planes.
Refinement. Refinement of *F*^2^ against ALL reflections. The weighted *R*-factor *wR* and goodness of fit *S* are based on *F*^2^, conventional *R*-factors *R* are based on *F*, with *F* set to zero for negative *F*^2^. The threshold expression of *F*^2^ > σ(*F*^2^) is used only for calculating *R*-factors(gt) *etc*. and is not relevant to the choice of reflections for refinement. *R*-factors based on *F*^2^ are statistically about twice as large as those based on *F*, and *R*- factors based on ALL data will be even larger.

### Fractional atomic coordinates and isotropic or equivalent isotropic displacement parameters (Å^2^)

**Table:**

	*x*	*y*	*z*	*U*_iso_*/*U*_eq_	
C1	0.3069 (4)	1.3641 (3)	0.4580 (4)	0.0893 (11)	
H1A	0.2011	1.3243	0.4505	0.134*	
H1B	0.3160	1.4451	0.4379	0.134*	
H1C	0.3651	1.3846	0.5413	0.134*	
C2	0.3654 (4)	1.2701 (3)	0.3728 (3)	0.0611 (8)	
H2A	0.3083	1.2498	0.2881	0.073*	
H2B	0.4726	1.3091	0.3800	0.073*	
C3	0.3773 (3)	1.0444 (2)	0.3310 (2)	0.0405 (6)	
C4	0.3423 (3)	0.9266 (2)	0.37091 (19)	0.0382 (5)	
C5	0.3652 (3)	0.8068 (3)	0.3117 (2)	0.0436 (6)	
C6	0.4214 (4)	0.7543 (3)	0.2014 (2)	0.0621 (8)	
H6A	0.4101	0.8081	0.1494	0.093*	
H6B	0.3631	0.6632	0.1563	0.093*	
H6C	0.5276	0.7573	0.2269	0.093*	
C7	0.2826 (2)	0.9094 (2)	0.47482 (18)	0.0346 (5)	
C8	0.2770 (3)	0.7808 (2)	0.46902 (19)	0.0388 (5)	
C9	0.2328 (3)	0.9897 (2)	0.5730 (2)	0.0404 (6)	
C10	0.1892 (3)	0.7846 (2)	0.6342 (2)	0.0402 (6)	
C11	0.1584 (3)	0.9935 (2)	0.76523 (19)	0.0372 (5)	
C12	0.0208 (3)	1.0263 (2)	0.7601 (2)	0.0433 (6)	
H12	−0.0514	1.0000	0.6847	0.052*	
C13	−0.0090 (3)	1.0983 (3)	0.8672 (2)	0.0486 (6)	
H13	−0.1013	1.1213	0.8653	0.058*	
C14	0.0991 (3)	1.1353 (2)	0.9765 (2)	0.0480 (6)	
C15	0.2366 (3)	1.1045 (3)	0.9847 (2)	0.0512 (7)	
H15	0.3080	1.1311	1.0605	0.061*	
C16	0.2661 (3)	1.0329 (2)	0.8772 (2)	0.0442 (6)	
H16	0.3591	1.0110	0.8798	0.053*	
C17	0.1711 (3)	0.6040 (2)	0.7262 (2)	0.0482 (6)	
H17	0.1861	0.5517	0.6475	0.058*	
C18	0.0384 (3)	0.5200 (3)	0.7535 (3)	0.0711 (9)	
H18A	−0.0525	0.4956	0.6862	0.085*	
H18B	0.0175	0.5719	0.8286	0.085*	
C19	0.0750 (4)	0.3927 (3)	0.7692 (3)	0.0855 (11)	
H19A	−0.0089	0.3431	0.7918	0.103*	
H19B	0.0843	0.3362	0.6910	0.103*	
C22	0.3165 (3)	0.6368 (3)	0.8250 (3)	0.0721 (9)	
H22A	0.3063	0.6937	0.9027	0.087*	
H22B	0.4009	0.6863	0.8028	0.087*	
C21	0.3524 (4)	0.5105 (4)	0.8417 (4)	0.0887 (11)	
H21A	0.3757	0.4594	0.7675	0.106*	
H21B	0.4423	0.5356	0.9100	0.106*	
F1	0.0682 (2)	1.20612 (17)	1.08220 (14)	0.0762 (5)	
N1	0.1902 (2)	0.91680 (18)	0.65370 (16)	0.0378 (5)	
N2	0.2326 (2)	0.71226 (19)	0.54199 (17)	0.0446 (5)	
N3	0.1374 (3)	0.7269 (2)	0.71300 (19)	0.0498 (6)	
H3	0.117 (3)	0.781 (3)	0.774 (2)	0.060*	
O1	0.3480 (2)	1.14890 (17)	0.40502 (15)	0.0509 (5)	
O2	0.4260 (2)	1.04606 (19)	0.24179 (15)	0.0588 (5)	
O3	0.32637 (19)	0.71581 (16)	0.37123 (14)	0.0467 (4)	
O4	0.2239 (2)	1.10393 (18)	0.59732 (17)	0.0639 (6)	
C20	0.2211 (4)	0.4244 (3)	0.8669 (3)	0.0785 (10)	
H20A	0.2073	0.4705	0.9472	0.094*	
H20B	0.2447	0.3414	0.8694	0.094*	

### Atomic displacement parameters (Å^2^)

**Table:**

	*U*^11^	*U*^22^	*U*^33^	*U*^12^	*U*^13^	*U*^23^
C1	0.116 (3)	0.060 (2)	0.113 (3)	0.040 (2)	0.051 (2)	0.036 (2)
C2	0.078 (2)	0.0520 (18)	0.0698 (18)	0.0230 (15)	0.0288 (15)	0.0349 (15)
C3	0.0412 (13)	0.0461 (15)	0.0371 (12)	0.0116 (11)	0.0116 (10)	0.0170 (11)
C4	0.0408 (13)	0.0407 (14)	0.0353 (11)	0.0116 (11)	0.0115 (10)	0.0144 (10)
C5	0.0492 (15)	0.0439 (15)	0.0394 (12)	0.0130 (12)	0.0139 (11)	0.0143 (11)
C6	0.087 (2)	0.0552 (18)	0.0517 (15)	0.0236 (16)	0.0370 (15)	0.0135 (13)
C7	0.0377 (12)	0.0329 (13)	0.0336 (11)	0.0083 (10)	0.0088 (9)	0.0128 (9)
C8	0.0445 (13)	0.0371 (13)	0.0334 (11)	0.0116 (11)	0.0092 (10)	0.0095 (10)
C9	0.0524 (15)	0.0373 (14)	0.0413 (12)	0.0160 (11)	0.0189 (11)	0.0208 (11)
C10	0.0493 (14)	0.0361 (14)	0.0413 (12)	0.0132 (11)	0.0141 (11)	0.0191 (11)
C11	0.0491 (14)	0.0311 (13)	0.0392 (12)	0.0116 (11)	0.0180 (10)	0.0187 (10)
C12	0.0499 (15)	0.0432 (14)	0.0465 (13)	0.0154 (12)	0.0194 (11)	0.0233 (11)
C13	0.0530 (16)	0.0496 (16)	0.0607 (16)	0.0240 (13)	0.0294 (13)	0.0286 (13)
C14	0.0699 (18)	0.0384 (14)	0.0444 (13)	0.0172 (13)	0.0284 (12)	0.0167 (11)
C15	0.0608 (17)	0.0560 (17)	0.0397 (13)	0.0137 (14)	0.0151 (12)	0.0201 (12)
C16	0.0469 (14)	0.0488 (15)	0.0457 (13)	0.0179 (12)	0.0162 (11)	0.0225 (12)
C17	0.0732 (18)	0.0363 (14)	0.0472 (13)	0.0219 (13)	0.0229 (12)	0.0224 (11)
C18	0.066 (2)	0.0461 (17)	0.107 (2)	0.0083 (14)	0.0117 (17)	0.0453 (18)
C19	0.094 (3)	0.0490 (19)	0.118 (3)	0.0104 (17)	0.013 (2)	0.049 (2)
C22	0.064 (2)	0.067 (2)	0.094 (2)	0.0068 (16)	0.0143 (17)	0.0515 (18)
C21	0.083 (2)	0.095 (3)	0.114 (3)	0.033 (2)	0.017 (2)	0.072 (2)
F1	0.1084 (14)	0.0745 (12)	0.0571 (9)	0.0383 (10)	0.0431 (9)	0.0164 (8)
N1	0.0501 (12)	0.0334 (11)	0.0378 (9)	0.0148 (9)	0.0169 (8)	0.0176 (8)
N2	0.0613 (13)	0.0339 (11)	0.0444 (11)	0.0145 (10)	0.0197 (10)	0.0164 (9)
N3	0.0759 (16)	0.0355 (13)	0.0520 (12)	0.0212 (11)	0.0302 (11)	0.0228 (10)
O1	0.0728 (12)	0.0399 (10)	0.0524 (10)	0.0168 (9)	0.0321 (9)	0.0228 (8)
O2	0.0806 (14)	0.0645 (13)	0.0517 (10)	0.0286 (10)	0.0368 (10)	0.0311 (9)
O3	0.0627 (11)	0.0374 (10)	0.0440 (9)	0.0158 (8)	0.0218 (8)	0.0128 (8)
O4	0.1135 (16)	0.0423 (11)	0.0695 (12)	0.0402 (11)	0.0578 (11)	0.0346 (9)
C20	0.104 (3)	0.070 (2)	0.086 (2)	0.032 (2)	0.0246 (19)	0.0550 (19)

### Geometric parameters (Å, °)

**Table:**

C1—C2	1.463 (4)	C12—C13	1.375 (3)
C1—H1A	0.9600	C12—H12	0.9300
C1—H1B	0.9600	C13—C14	1.365 (4)
C1—H1C	0.9600	C13—H13	0.9300
C2—O1	1.449 (3)	C14—F1	1.363 (3)
C2—H2A	0.9700	C14—C15	1.365 (4)
C2—H2B	0.9700	C15—C16	1.377 (3)
C3—O2	1.208 (3)	C15—H15	0.9300
C3—O1	1.318 (3)	C16—H16	0.9300
C3—C4	1.472 (3)	C17—N3	1.464 (3)
C4—C5	1.357 (3)	C17—C22	1.505 (4)
C4—C7	1.461 (3)	C17—C18	1.509 (4)
C5—O3	1.384 (3)	C17—H17	0.9800
C5—C6	1.478 (3)	C18—C19	1.531 (4)
C6—H6A	0.9600	C18—H18A	0.9700
C6—H6B	0.9600	C18—H18B	0.9700
C6—H6C	0.9600	C19—C20	1.504 (4)
C7—C8	1.361 (3)	C19—H19A	0.9700
C7—C9	1.432 (3)	C19—H19B	0.9700
C8—N2	1.343 (3)	C22—C21	1.522 (4)
C8—O3	1.362 (3)	C22—H22A	0.9700
C9—O4	1.207 (3)	C22—H22B	0.9700
C9—N1	1.446 (3)	C21—C20	1.498 (5)
C10—N2	1.313 (3)	C21—H21A	0.9700
C10—N3	1.352 (3)	C21—H21B	0.9700
C10—N1	1.377 (3)	N3—H3	0.86 (3)
C11—C12	1.378 (3)	C20—H20A	0.9700
C11—C16	1.383 (3)	C20—H20B	0.9700
C11—N1	1.442 (3)		
			
C2—C1—H1A	109.5	C14—C15—C16	117.9 (2)
C2—C1—H1B	109.5	C14—C15—H15	121.1
H1A—C1—H1B	109.5	C16—C15—H15	121.1
C2—C1—H1C	109.5	C15—C16—C11	120.4 (2)
H1A—C1—H1C	109.5	C15—C16—H16	119.8
H1B—C1—H1C	109.5	C11—C16—H16	119.8
O1—C2—C1	107.8 (2)	N3—C17—C22	110.7 (2)
O1—C2—H2A	110.1	N3—C17—C18	110.7 (2)
C1—C2—H2A	110.1	C22—C17—C18	111.0 (2)
O1—C2—H2B	110.1	N3—C17—H17	108.1
C1—C2—H2B	110.1	C22—C17—H17	108.1
H2A—C2—H2B	108.5	C18—C17—H17	108.1
O2—C3—O1	123.8 (2)	C17—C18—C19	110.6 (3)
O2—C3—C4	124.9 (2)	C17—C18—H18A	109.5
O1—C3—C4	111.31 (19)	C19—C18—H18A	109.5
C5—C4—C7	106.1 (2)	C17—C18—H18B	109.5
C5—C4—C3	123.0 (2)	C19—C18—H18B	109.5
C7—C4—C3	130.9 (2)	H18A—C18—H18B	108.1
C4—C5—O3	110.5 (2)	C20—C19—C18	111.8 (3)
C4—C5—C6	134.8 (2)	C20—C19—H19A	109.2
O3—C5—C6	114.8 (2)	C18—C19—H19A	109.2
C5—C6—H6A	109.5	C20—C19—H19B	109.2
C5—C6—H6B	109.5	C18—C19—H19B	109.2
H6A—C6—H6B	109.5	H19A—C19—H19B	107.9
C5—C6—H6C	109.5	C17—C22—C21	111.6 (3)
H6A—C6—H6C	109.5	C17—C22—H22A	109.3
H6B—C6—H6C	109.5	C21—C22—H22A	109.3
C8—C7—C9	117.43 (19)	C17—C22—H22B	109.3
C8—C7—C4	105.55 (19)	C21—C22—H22B	109.3
C9—C7—C4	137.0 (2)	H22A—C22—H22B	108.0
N2—C8—C7	130.9 (2)	C20—C21—C22	111.9 (3)
N2—C8—O3	117.8 (2)	C20—C21—H21A	109.2
C7—C8—O3	111.33 (19)	C22—C21—H21A	109.2
O4—C9—C7	130.5 (2)	C20—C21—H21B	109.2
O4—C9—N1	118.1 (2)	C22—C21—H21B	109.2
C7—C9—N1	111.35 (19)	H21A—C21—H21B	107.9
N2—C10—N3	119.0 (2)	C10—N1—C11	119.56 (17)
N2—C10—N1	123.2 (2)	C10—N1—C9	124.19 (19)
N3—C10—N1	117.7 (2)	C11—N1—C9	116.10 (17)
C12—C11—C16	120.3 (2)	C10—N2—C8	112.82 (19)
C12—C11—N1	120.03 (19)	C10—N3—C17	122.8 (2)
C16—C11—N1	119.7 (2)	C10—N3—H3	114.2 (19)
C13—C12—C11	119.5 (2)	C17—N3—H3	118.7 (18)
C13—C12—H12	120.2	C3—O1—C2	118.03 (18)
C11—C12—H12	120.2	C8—O3—C5	106.58 (18)
C14—C13—C12	118.9 (2)	C21—C20—C19	111.4 (2)
C14—C13—H13	120.5	C21—C20—H20A	109.3
C12—C13—H13	120.5	C19—C20—H20A	109.3
F1—C14—C13	118.4 (2)	C21—C20—H20B	109.3
F1—C14—C15	118.5 (2)	C19—C20—H20B	109.3
C13—C14—C15	123.0 (2)	H20A—C20—H20B	108.0
			
O2—C3—C4—C5	1.6 (4)	N3—C17—C22—C21	179.1 (2)
O1—C3—C4—C5	−178.7 (2)	C18—C17—C22—C21	55.7 (4)
O2—C3—C4—C7	−179.5 (2)	C17—C22—C21—C20	−54.7 (4)
O1—C3—C4—C7	0.2 (3)	N2—C10—N1—C11	−172.6 (2)
C7—C4—C5—O3	−0.8 (3)	N3—C10—N1—C11	8.9 (3)
C3—C4—C5—O3	178.38 (19)	N2—C10—N1—C9	2.7 (4)
C7—C4—C5—C6	179.5 (3)	N3—C10—N1—C9	−175.9 (2)
C3—C4—C5—C6	−1.3 (4)	C12—C11—N1—C10	−107.4 (2)
C5—C4—C7—C8	0.7 (2)	C16—C11—N1—C10	72.2 (3)
C3—C4—C7—C8	−178.3 (2)	C12—C11—N1—C9	77.0 (3)
C5—C4—C7—C9	−179.3 (3)	C16—C11—N1—C9	−103.4 (2)
C3—C4—C7—C9	1.6 (4)	O4—C9—N1—C10	177.3 (2)
C9—C7—C8—N2	−0.6 (4)	C7—C9—N1—C10	−3.6 (3)
C4—C7—C8—N2	179.4 (2)	O4—C9—N1—C11	−7.3 (3)
C9—C7—C8—O3	179.58 (18)	C7—C9—N1—C11	171.78 (18)
C4—C7—C8—O3	−0.4 (2)	N3—C10—N2—C8	178.2 (2)
C8—C7—C9—O4	−178.6 (3)	N1—C10—N2—C8	−0.3 (3)
C4—C7—C9—O4	1.4 (5)	C7—C8—N2—C10	−0.7 (4)
C8—C7—C9—N1	2.5 (3)	O3—C8—N2—C10	179.16 (19)
C4—C7—C9—N1	−177.5 (2)	N2—C10—N3—C17	19.9 (4)
C16—C11—C12—C13	−0.2 (3)	N1—C10—N3—C17	−161.5 (2)
N1—C11—C12—C13	179.3 (2)	C22—C17—N3—C10	90.1 (3)
C11—C12—C13—C14	−0.1 (3)	C18—C17—N3—C10	−146.4 (3)
C12—C13—C14—F1	−179.7 (2)	O2—C3—O1—C2	4.3 (3)
C12—C13—C14—C15	0.2 (4)	C4—C3—O1—C2	−175.4 (2)
F1—C14—C15—C16	180.0 (2)	C1—C2—O1—C3	172.4 (2)
C13—C14—C15—C16	0.1 (4)	N2—C8—O3—C5	−179.9 (2)
C14—C15—C16—C11	−0.4 (4)	C7—C8—O3—C5	0.0 (2)
C12—C11—C16—C15	0.5 (3)	C4—C5—O3—C8	0.5 (3)
N1—C11—C16—C15	−179.1 (2)	C6—C5—O3—C8	−179.7 (2)
N3—C17—C18—C19	−179.1 (2)	C22—C21—C20—C19	53.8 (4)
C22—C17—C18—C19	−55.8 (4)	C18—C19—C20—C21	−54.4 (4)
C17—C18—C19—C20	55.4 (4)		

### Hydrogen-bond geometry (Å, °)

**Table:**

*D*—H···*A*	*D*—H	H···*A*	*D*···*A*	*D*—H···*A*
C17—H17···N2	0.98	2.41	2.813 (3)	104
C6—H6A···O2	0.96	2.45	3.039 (3)	120
C20—H20B···Cg3^i^	0.97	2.97	3.820 (4)	147
C14—F···Cg3^ii^	1.363 (3)	3.358 (2)	3.732 (3)	95
C3—O2···Cg2^iii^	1.208 (3)	3.309 (2)	3.409 (3)	84

Symmetry codes: (i) *x*, *y*−1, *z*; (ii) −*x*, −*y*+2, −*z*+2; (iii) −*x*+1, −*y*+2, −*z*+1.
